# Copeptin in Growth Hormone-Treated Patients

**DOI:** 10.3390/jcm11195510

**Published:** 2022-09-20

**Authors:** Anna Sjöström, Simona I. Chisalita, Charlotte Höybye

**Affiliations:** 1 Department of Clinical Chemistry, Karolinska University Hospital, 171 76 Stockholm, Sweden; 2 Department of Molecular Medicine and Surgery, Karolinska Institutet, 171 76 Stockholm, Sweden; 3 Department of Health, Medicine and Caring Sciences, Faculty of Medicine and Health Sciences, Linköping University, 581 83 Linköping, Sweden; 4 Department of Endocrinology, Linköping University Hospital, 581 85 Linköping, Sweden; 5 Department of Endocrinology, Karolinska University Hospital, 171 76 Stockholm, Sweden

**Keywords:** growth hormone, copeptin, growth hormone deficiency, arginine-vasopressin

## Abstract

Growth hormone (GH) changes body composition, including increasing body water. GH is known to have an anti-natriuretic effect in the kidney, but little is known of its effect on arginine-vasopressin (AVP) release. We studied the effect of GH on AVP release by measurement of copeptin, a fragment from the same precursor protein, in GH-treated patients with GH deficiency. The study was designed as a retrospective cohort study of biobank samples from 34 patients substituted with GH between 1999 and 2004. Copeptin and insulin-like growth factor 1 (IGF-1) results were compared with previously obtained data. An increase in IGF-1 and copeptin was seen at 3 and 6 months’ treatment compared to baseline. Between the 3 and 6 months follow up, copeptin levels were stable. There was a difference in HbA1c between 3 and 6 months (*p* < 0.01) and between baseline and 6 months (*p* = 0.042), with higher levels at 6 months. In addition, LDL levels were lower at the 6 months follow up (*p* = 0.046). The waist circumference at 3 months was lower (*p* = 0.02). To conclude, three months of GH treatment increased the levels of copeptin and the increase remained at 6 months. This could be a compensatory mechanism balancing the anti-natriuretic effect of GH treatment seen in previous studies.

## 1. Introduction

Copeptin is a 39-amino acid glycosylated peptide and a part of the same precursor protein as arginine-vasopressin (AVP, antidiuretic hormone). It is synthesized in the hypothalamus and stored in the posterior lobe of the pituitary [1]. During the transport from the hypothalamus to the pituitary, enzymatic cleavage occurs, and the precursor protein is split to the final products, separating copeptin and AVP in their neurosecretory granulae. When osmotic or baroreceptors sense low osmotic level or low blood pressure, AVP and copeptin are released, in an equimolar amount, to the blood stream. AVP stimulates aquaporins in the kidney tubule to reabsorb water from the urine, hence lowering osmolality and elevating blood pressure [2,3].

Due to a habitual low concentration, the small size of the bioactive polypeptide, and a rapid degradation in the blood, AVP is difficult to analyze. The more stable, slightly bigger, and hormonally inactive copeptin is therefore an interesting biomarker replacing measurement of AVP [4].

Growth hormone (GH) is synthesized in the anterior lobe of the pituitary and is released in a pulsatile manner. The pulsatile and irregular release of GH makes it an erratic biomarker and in routine medicine insulin-like growth factor 1 (IGF-1) is used instead. IGF-1 is mainly produced in the liver in response to stimulation by GH and is a mediator of its metabolic effects [5]. IGF-I has a more stable 24 h level that decreases with age [6].

In adults, GH deficiency can be caused by various conditions, most frequently a pituitary adenoma or its treatment but also more rare disorders like autoimmune hypophysitis [7]. Symptoms of GH deficiency in adults include central obesity, reduced muscle mass and poor quality of life, unfavorable blood lipids as well as increased glucose levels [8,9,10]. Moreover, GH deficiency is associated with dehydration [11].

Treatment with GH in GH-deficient patients has in several studies shown improved body composition, muscle mass and strength, exercise capacity, cardiovascular risk factors, glucose and lipid profile, bone metabolism, and quality of life [12]. Side effects derived from water retention caused by GH occur in 5–18% of patients. The fluid retention clinically manifests as edema, arthralgias, myalgias, and carpal tunnel syndrome, and usually improves after GH dose reduction [13]. It is well known that GH treatment increases blood glucose and might cause diabetes in some patients. There are no long-term data that GH treatment increases the risk for cardiovascular disease or cancer [14,15].

The underlying etiology to the volume expansion during GH treatment is not fully understood but increased levels of renin and aldosterone have been found. In addition, AVP could potentially be high in response to the hypovolemic state and higher osmolality seen in GH deficiency. To our knowledge, levels of AVP during long-term GH treatment have not been studied previously and our aim was to investigate levels of AVP by measurements of copeptin in bio-bank samples from GH-deficient patients during 6 months of GH treatment.

## 2. Patients and Methods

The study was approved by the Swedish Ethical Authority (D-nr 2018-1243-31/1) and no study-related activity was performed before receiving patients’ informed consent.

In patients with confirmed GH deficiency treated with GH during 1999–2004, serum was sampled for bio-bank storage at the patient’s routine visits to the clinic. Based on availability of bio-bank samples, a cohort of 34 patients with GHD was identified.

All patients had confirmed GH deficiency according to consensus guidelines. The patients were treated once daily with subcutaneous injection of GH at bedtime. Doses were individually titrated to IGF-I levels in age-matched healthy subjects and the starting dose was between 0.1 and 0.2 mg/day. Clinical examinations and sampling of fasting routine blood tests were performed at scheduled visits to the clinic and in addition 2 mL serum was stored in a biobank at −70 degrees.

The patients were enrolled in two post-marketing observational databases of GH treatment in adults with GHD, NordiNet (NovoNordisk), and KIMS (Pfizer). Data on demography (age at baseline, gender, smoking), weight, BMI, waist circumference, blood pressure, fasting blood glucose, HbA1c, total, LDL- and HDL-cholesterol, triglycerides, IGF-I, concomitant medication, clinical events, and co-morbidities were retrieved from the study databases and the patients’ medical records at baseline and during GH treatment (see Supplementary Table S1 for laboratory reference intervals). Three patients were on stable treatment with statins and one woman was treated with low-dose estrogen.

One patient with ADH deficiency was excluded, resulting in a final cohort of 33 patients.

In serum samples from the biobank, copeptin was analysed before and after approximately 3 and 6 months of GH treatment.

### 2.1. Anthropometric Methods

Physical examination included measurements of weight and waist circumferences. Waist circumferences were measured in standing position. Waist circumference was measured halfway between the costal edge and iliac crest and cut-off points for obesity were set to 88 cm in female and 102 cm in male patients. Body mass index (BMI) was calculated as weight divided by the square height, kg/m^2^. BMI from 18.5 to 25 kg/m^2^ was defined as normal, between 25 and 30 kg/m^2^ as overweight and above 30 kg/m^2^ as obese, according to WHO criteria.

### 2.2. Assays

Fasting blood glucose was determined with the glucose oxidase method using a standard glucose analyzer (YSI, Inc., Yellow Springs, OH, USA). Normal fasting blood glucose < 6.1 mmol/L. HbA_1C_ was analyzed using the Mono-S method and IFCC, normal HbA1C < 42 mmol/L. Results from the Mono-S method were converted to mmol/mol. Sodium was measured by ion selective electrodes (Hitachi 911).

Serum cholesterol and triglycerides were measured with colorimetric methods (Vitos 900) and high-density lipoprotein (HDL) with direct calorimetry (Hitachi 911). LDL-cholesterol concentration was calculated according to Friedewald’s formula. Serum IGF-I levels were measured using a solid-phase, enzyme-labelled chemiluminescent immunometric assay (IMMULITE 2000, Siemens, Munich, Germany).

Copeptin was analyzed with Brahms Cryptor at Linköping university hospital.

### 2.3. Statistical Analysis

Statistical analysis was performed using GraphPad Prism version 5 and IBM SPSS statistics V.23.0 (binary regression analysis, IBM, Armonk, NY, USA). Data are presented as medians, interquartile range or percent. Wilcoxon matched-pairs signed rank test was used to assess differences over time regarding variables. Mann–Whitney U test was used to assess differences between gender. Data regarding the association of isolated growth hormone deficiency with copeptin levels at baseline, 3 and 6 months follow up were analyzed with binary logistic regression to estimate adjusted ORs for the single outcome variable identified above. Statistical significance was set to *p* < 0.05.

## 3. Results

Median age at baseline was 58 years and there was an even sex distribution, see Table 1. The median patient was slightly overweight. Three patients were smokers.

Eighteen patients had previously been treated for non-functioning pituitary adenomas (NFPA), 2 patients for GH-producing adenoma, 4 patients for ACTH-producing adenoma, 6 for prolactinoma, and 8 patients for other diseases in the area. The patients had been treated with pituitary surgery, fractionated radiotherapy, Gamma Knife Radiosurgery and pharmacological treatments and they were all cured or in remission for their pituitary disease when GH treatment was initiated.

Five patients had no other pituitary deficiencies than GH deficiency, 9 had one pituitary deficiency in addition to GH deficiency, 13 patients two additional deficiencies, and 7 patients three additional deficiencies. Replacements for pituitary deficiencies other than GHD had been stable for many years.

All had normal kidney function. Several patients were on pain-relieving and anti-depressive medication.

The patients without any deficiencies other than GH (N = 5) did not show any different copeptin pattern, only less pronounced, than the total cohort.

The patients started with a dose of GH 0.2 mg/day (IQR 0.1–0.2) and continued to have 0.2 mg/day in median but with IQR 0.2–0.3 at both 3 and 6 months follow up. In general, the copeptin levels were low within normal range and increased within normal range during GH treatment. IGF-1 increased from subnormal to normal levels, see Table 2.

**Figure 1:**
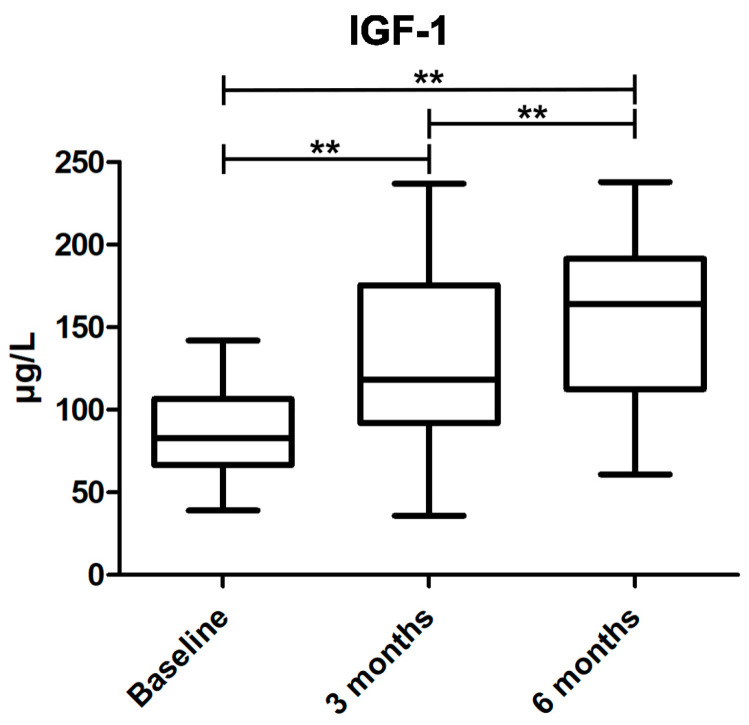
Insulin-like growth factor 1 (IGF-1) levels over time. Box plots with interquartile range of IGF-1 levels at baseline and 3 and 6 months follow up. ** indicates significance *p* < 0.01.

All, except one patient, increased in IGF-1 levels during the treatment period and the Wilcoxon matched-pairs signed rank test showed an increase between baseline and 3 months, as well as 6 months, treatment but also an increase between 3 months and 6 months treatment (*p* < 0.01 for all periods), see Figure 1.

**Figure 2:**
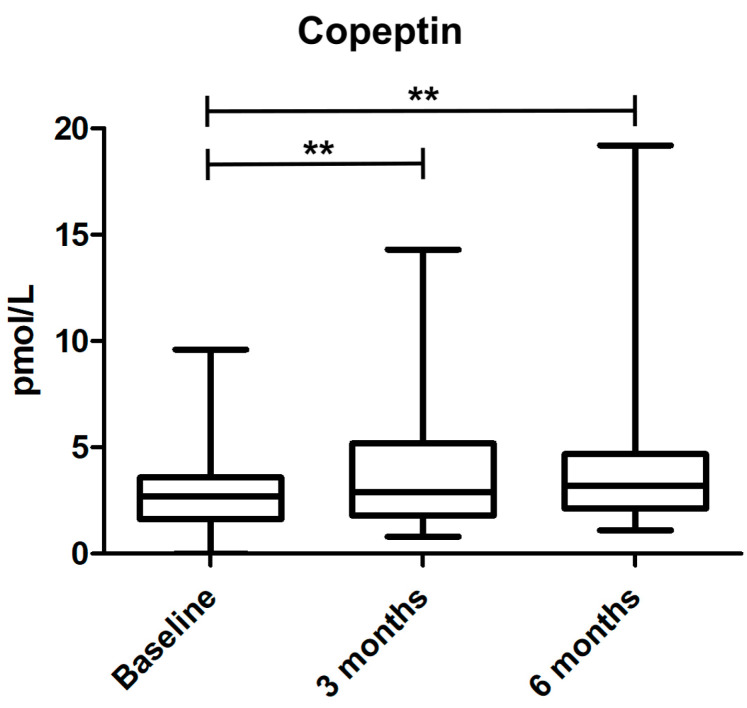
Copeptin levels over time. Box plots with interquartile range of copeptin levels at baseline and 3 and 6 months follow up. ** indicates significance *p* < 0.01.

Statistical analysis, with Wilcoxon matched-pairs signed rank test, showed an increase of copeptin levels during the treatment period between baseline and 3 months and baseline and 6 months (*p* < 0.01 for both periods) but no further increase between 3 and 6 months (*p* = 0.91), see Figure 2.

Most of the patients (n = 29) increased in copeptin levels with treatment but in four patients copeptin decreased (median of 3.1 pmol/L at baseline to 2.3 pmol/L at 6 months treatment, in median with a decrease of 14%) during treatment. These four patients all had an above 100% increase of IGF-1 between baseline and 6 months’ treatment, indicating a good treatment adherence. Removing the four patients from the statistical analysis did not change the significance for the increase in copeptin and IGF-1.

There were no significant differences between men and women regarding levels of IGF-1 and copeptin, using the Mann–Whitney U test, except for copeptin levels at 3 months (*p* < 0.01).

The other measured biomarkers showed a statistically significant difference in HbA1c between 3 and 6 months (*p* < 0.01) and between baseline and 6 months (*p* = 0.042). LDL levels were lower at the 6 months follow up (*p* = 0.046). There was no difference between baseline, 3 months, and 6 months regarding triglycerides, HDL, total cholesterol, sodium, or glucose. The results for the patients on statins (N = 3) and estrogen (N = 1) did not differ from the other patients.

Looking at clinical parameters, the only difference was between waist circumference at baseline and at three months follow up (*p* = 0.02). No differences between baseline and follow ups were seen in pulse, blood pressure, weight, or BMI.

Five of the 33 patients included in the study had isolated growth hormone deficiency. In this group, the association between isolated GH deficiency and copeptin levels at baseline and at the 3 and 6 months follow up was tested in univariate binary regression models. No significant association was found between isolated GH deficiency and copeptin levels at the baseline and 3 and 6 months follow ups (OR 0.69 (CI 0.33–1.45), *p* = 0.33; OR 0.88 (CI 0.55–1.39), *p* = 0.59 and OR 0.79 (0.43–1.45), *p*= 0.45, respectively).

## 4. Discussion

In this study of 33 patients treated with GH for 6 months, we found a significant increase in IGF-I and in copeptin after 3 months that remained at 6 months. HbA1c increased and LDL decreased from baseline to the 6 months follow up while there was no change in blood pressure or pulse during treatment. Weight and BMI increased non-significantly, while waist circumference decreased. Men were found to have higher copeptin levels at the 3 months follow up, but due to the small sample size it is not feasible to make any assumptions from this.

Despite the low GH doses administered in our cohort, IGF-I levels increased and simultaneously we observed the expected increase in HbA1c. The increase in HbA1c was modest and well below the upper reference level. The increase in HbA1c during GH treatment is well known also with low doses of GH [16]. In accordance with previous studies, we found a decrease in LDL cholesterol, while no differences in effects between men and women were noticed.

In adults with GH deficiency, total body water, lean body mass, and extracellular and intracellular water are reduced. The chronic hypovolemic state may alter regulatory mechanisms of fluid balance, which is then normalized when GH treatment corrects the hypovolemia inducing sodium and fluid retention [17]. Previous studies have indicated that AVP stimulates release of GH but little is known about an opposite effect [18]. The well known sodium retention and hypertension seen in acromegaly and GH-treated patients have been investigated in several studies, and have been explained to be caused by an anti-natriuretic effect on the aquaporin channels in the kidney [19,20]. In rats, activation of a renal Na^+^-K^+^-2Cl^−^ transporter is seen after GH stimulation, increasing reabsorption of sodium in the kidney [21]. The relationship between extracellular water and GH is not linear. In the acute phase, sodium reabsorption and volume expansion occur, but over time, during continued GH administration, there is a normalization of urinary sodium but with a consistent volume expansion [22]. This could be the result of inhibition of sodium transport by higher concentrations of GH or by unknown renal changes during chronic GH exposure [23,24]. In addition to an increased volume expansion, increased levels of renin and aldosterone have been found in GH-treated patients [19,25].

In the current cohort, treatment with GH increased copeptin/AVP levels. Levels of copeptin were low-normal at baseline. Considering that long-term GH deficiency results in a chronic hypovolemic state, high levels of AVP/copeptin would be expected. The hypovolemia is clinically usually not very pronounced, and together with the chronic state, a different steady state is presumably present with an activated RAAS system and downregulated AVP secretion. When GH treatment is initiated, a new steady state is obtained with downregulation of the RAAS system and an increase in the AVP/copeptin secretion to decrease sodium and osmolality. This is speculation, but in our cohort AVP/copeptin levels increased at 3 months and remained at the higher levels at 6 months, indicating that a desired regaining of fluid by AVP/copeptin occurs early and then remains unchanged. The increase in AVP/copeptin was significant but supranormal levels were not noted, probably because the baseline hypovolemia was not very pronounced clinically.

Previous studies have not shown an increase of AVP, which may be caused by its short half-life in vivo and its tendency to be affected by preanalytical errors [19]. In our study, most of the patients’ copeptin level increased but in a subgroup it did not despite an increase in IGF-I. We do not have an explanation for this, but maybe the hypovolemia in those patients was not present or other factors like medication or lifestyle factors played a role. At any rate, our data suggest that AVP plays a role in normalizing fluid balance in patients with GH deficiency. In line with the relatively low levels of copeptin in this cohort, none of them received high doses of GH or complained of edema or other symptoms of fluid retention.

The correlation of IGF1 and copeptin was poor, also after correction for age, which probably is an effect of the very varying, individual levels of IGF-1. Moreover, an association between isolated GH deficiency and copeptin was not seen, which could be caused by the small sample size.

The strength of this study is the careful prospective sampling of serum during GH treatment. However, our study has limitations. A control, or placebo-treated, group was not established as the serum samples were collected many years ago and only from patients with GHD, which made it difficult to identify a proper control group as of today. The cohort was small and only followed for 6 months; thus, the effect of long-term GH treatment on copeptin levels is unknown. Another limitation of the study is that patients were included based on the availability of serum samples, which might have selected a cohort of healthier patients. An obvious limitation to the study is the long storage time of the biobank samples, and even though the samples were stored in a -70-degree freezer, degradation cannot be excluded. Furthermore, all patients had an anterior pituitary insufficiency and maybe a reduced function of the pituitary; a reduced function of the posterior pituitary might have been present.

## 5. Conclusions

In conclusion, our study of 33 patients with GH deficiency showed, for the first time, an increase in AVP/copeptin during GH treatment, indicating a role of AVP in normalizing the hypovolemia of adult GH-deficiency syndrome. Our results also offer an additional explanation for the fluid retention caused by high GH doses. Our study has limitations and systematic, prospective studies are needed to confirm our results.

## Figures and Tables

**Table 1:** Baseline descriptive variables of 33 patients with growth hormone (GH) deficiency.

Descriptive Characteristics of the Cohort	
Age, years	58 (54–65)
Women, N (%)	17 (50)
Weight, kg	79 (70–92)
Waist, cm	95 (89.5–107.5)
Body mass index (BMI), kg/m^2^	26.7 (24.2–30.5)
Smoking, N (%)	3 (9)
Pulse, bpm	64 (60–73)
Diastolic blood pressure, mmHg	85 (80–90)
Systolic blood pressure, mmHg	135 (20–142.5)

Results are presented as median and interquartile range if not stated otherwise.

**Table 2:** Laboratory parameters over time of 33 patients with growth hormone (GH) deficiency.

Laboratory Parameters over Time	Baseline	3 Months	6 Months
IGF-1, µg/L	83 (66–107)	118 (92–175)	164 (113–192)
IGF-1 standard deviation score (SDS)	−1.1 (−1.6—0.7)	−0.2 (−0.9–1.0)	0.6 (−0.3–1.3)
Copeptin, pmol/L	2.7 (1.6–3.6)	2.9 (1.8–5.2)	3.1 (2.1–4.7)
Sodium, mmol/L	140 (138–141)	140 (139–142)	140 (138–140)
Glucose, mmol/L	4.6 (4.1–5.3)	4.8 (4.5–5.3)	4.8 (4.4–5.2)
Hba1c, mmol/mol	32 (28–38)	29 (26–38)	33 (28–38)
Total cholesterol, mmol/L	5.5 (4.7–6.4)	N/A	5.5 (4.7–6.4)
High density lipoprotein (HDL), mmol/L	1.3 (1.2–1.7)	N/A	1.2 (0.9–1.6)
Low density lipoprotein (LDL), mmol/L	3.2 (2.8–4.1)	N/A	3.1 (2.4–3.8)
Triglycerides, mmol/L	1.4 (1.1–1.9)	1.8 (1.0–2.2)	1.4 (1.1–1.9)

Results are presented as median and interquartile range.

## Data Availability

The data are not publicly available due to privacy and ethical restrictions. The data that support the findings of this study are available on request from the corresponding author.

## Supplementary Materials

The following supporting information can be downloaded at: https://www.mdpi.com/article/10.3390/jcm11195510/s1, Table S1: Laboratory reference intervals.
