# Supplementary material for: Copeptin in Growth Hormone-Treated Patients
Source: J Clin Med. 2022 Sep 20;11(19):5510. doi: 10.3390/jcm11195510 (PMC9570667; doi:10.3390/jcm11195510)
Supplement: Supplementary file 1 [file jcm-11-05510-s001.zip › jcm-1906279-supplementary.pdf]

Supplementary Table S1. Laboratory reference intervals.

Laboratory parameter	Reference intervals	
	Years	Reference range
IGF-1, μg/L	19-21	117-323
	22-24	99-289
	25-29	84-259
	30-34	71-234
	35-39	63-223
	40-44	58-219
	45-49	53-215
	50-54	48-209
	55-59	45-210
	60-64	43-220
	65-69	40-225
	70-79	35-216
	80-90	31-208
Copeptin, pmol/L	⩾18	<13.1
Sodium, mmol/L	⩾18	137-145
Glucose, mmol/L	⩾18	4.2-6.0
Hba1c, mmol/mol	< 50 år	27-42
	⩾ 50 år	31-46
Total cholesterol, mmol/L	18-30 år	2,9-6,1
	31-50 år	3,3-6,9
	⩾51 år	3,9-7,8
HDL, mmol/L	⩾18 år kvinnor	1,0-2,7
	⩾18 män	0,8-2,1
LDL, mmol/L	18-31 år	1,2-4,3
	31-50 år	1,4-4,7
	⩾51 år	2,0-5,3
Triglycerides, mmol/L	⩾18 år	0,45-2,6
